# Death of Dr. William Ring

**Published:** 1887-06

**Authors:** 


### *DEATH OF DR. WILLIAM RING.*

"One of Buffalo's most highly-esteemed physicians, Dr. William Ring, died April 21st, after an illness of only two days. Always a man of robust physique, he had probably never suffered a day's illness, with the exception of an attack of cholera in 1849, contracted in his frequent exposures during the epidemic in that year. This attack, however, predisposed him to bilious affections, from which he had suffered more or less ever since. On Sunday last he was taken with a severe attack of bilious colic, which on Monday developed into peritonitis, and it was then seen by Dr. E. T. Dorland and Dr. Charles Ring, who attended him, that nothing could be done to save his life. Apparently he did not realize his serious condition, and until a few hours of his end thought he would recover and live to accomplish more good. His last hours were not painful, and he passed away in peace.

"Dr. William Ring was born in De Ruyter, Madison County, on November 17, 1824. His father was Elihu Ring, a farmer, and his early education was gained principally at the country schools. Coming to Buffalo about 1847 to study medicine, he graduated from the Buffalo Medical College, then in the old Postoffice Building, on July 17, 1847, his diploma being the first to be issued by the college; he soon afterwards began practice in Buffalo, and with unusual success, his natural talent for the profession soon making itself apparent, and his faithful labors and the success attending them gaining him many friends and the respect of his fellow-practitioners. His life ever since had been that of a hard-working, charitable dispenser of good, and from the first to the last was largely spent among the poor, with whom his name became a familiar household word. His untiring work among this unremunerative class was never lessened during his lifetime. An intimate friend, in speaking on this subject last night, recalled a favorite expression of his—that he preferred to treat the poor, for God was their paymaster. Few men have stood higher in their profession and few have numbered as many firm friends among all classes of people. Since 1864 he had charge of the Providence Insane Asylum as medical superintendent; he was a member of the American Medical Association, the New York State Medical Association, the New York State Medical Society, president of the Erie County Medical Society, and of the Buffalo Medical and Surgical Association. About 1862 he was elected Supervisor for two terms; he was also physician at several public institutions at various periods of his life. No elective public positions were ever sought by him He was particularly interested and unusually successful in the treatment of the insane, and possessed a peculiar faculty in dealing with them. 'One must always treat an insane man honorably if he would succeed with him,' was one of his well-remembered expressions.

"Dr. Ring leaves two children, Charles and William G., both physicians, the former employed at the Almshouse.

"The Erie County Medical Society met, and Dr. E. T. Dorand was chosen to preside. He paid a fitting tribute to the departed, and was followed by Drs. John Cronyn, F. W. Bartlett, F. S. Crego, Edward Storck, P. H. Strong, John Hauenstein, J. B. Samo, and M. Hartwig, who gave interesting reminiscences of the deceased, and spoke with feeling of the qualities that made him a most honored member of the profession and won him so many friends in this community, as few men can boast Resolutions presented by a committee consisting of Drs. Cronyn, Strong and Samo, were adopted. The following members of the society acted as bearers at the funeral: Drs. P. H. Strong, Milan Baker, George N. Burwell, John Cronyn, J. B. Samo, William C. Phelps, Conrad Diehl, F. S. Crego, John Hauenstein and J. C. Greene.

"This tribute comes from the Sisters of Providence Asylum, to which Dr. Ring was a devoted physician for twenty-four years: 'His unbounded charity in various ways to the poor first drew the attention of the Sisters to him, and caused his appointment as the physician of the Providence Asylum when it was first opened. His fidelity and kindness in attending the place during almost a quarter of a century won for him the entire confidence of the Sisters and patients. As a physician he was very skillful and successful, and effected many cures. He was quiet, gentle and unassuming. He was ever prompt and willing to attend any call of distress, night or day. For his strict observance of the principles of honor, justice and veracity in all his dealings he was deservedly honored and revered. His many noble qualities endeared him so much to his numerous patients that his professional visits and cheerful words of admonition were a healing balm to many a suffering heart. The tears of those he served so well now follow him to the tomb as to the sacred resting-place of one who lived more to relieve his fellow men from affliction and to be acceptable to the Heavenly Physician than to gain wealth or glory here below. The highest honor and gratitude are due to Dr. Ring for his good works and eminent virtues.'"—*Buffalo Express.*

